# Nephron sparing surgery for renal tumors-comparison of off-clamp partial nephrectomy with hilar clamping

**DOI:** 10.12669/pjms.36.3.1533

**Published:** 2020

**Authors:** Imran Khan Jalbani, Syed Muhammad Nazim, Maria Ahmed, Farhat Abbas

**Affiliations:** 1 Dr. Imran Khan Jalbani, Aga Khan University, Karachi, Pakistan; 2 Dr. Syed Muhammad Nazim, Aga Khan University, Karachi, Pakistan; 3 Dr. Maria Ahmed, Resident General Surgery, University of Texas Southwestern, Dallas, TX 214-449-8350, USA; 4 Prof. Farhat Abbas, Aga Khan University, Karachi, Pakistan

**Keywords:** Clamping, Partial nephrectomy, Renal function, Renal tumor

## Abstract

**Background and Objective:**

Open partial nephrectomy (PN) is still considered gold standard procedure for T1 localized renal tumors. Conventional technique involves clamping of the renal artery with or without vein however, renal ischemia produces a certain level of damage to the kidneys. This study aims to investigate potential effect of off-clamp vs. hilar clamping PN on renal function.

**Methods:**

This is a retrospective cohort study of patients who underwent unilateral, open partial nephrectomy for renal tumors b/w January 2009 December 2016 at our institution. A total of 90 partial nephrectomies were performed of which 65 cases were eligible for analysis. Non clamping technique was used in 43 while clamp was applied in 22 patients. Variables studied were patients’ demographics, clinical variables, the laterality, tumors size and location, R.E.N.A.L nephrometry score, blood loss, tumor histology and surgical margins. Patients’ renal function (serum creatinine and eGFR) were determined pre-operatively, at 3 and 12 months follow up. Data was analyzed on SPSS v. 22.

**Results:**

Both the groups were comparable with regards to pre-operative renal function. Mean radiological size of tumor was 4.71±1.31 and 3.81±1.0 (0.003) in two groups respectively. Mean R.E.N.A.L nephrometry score was 6.1±1.5 in off-clamp group compared to 7.05 ± 1.7 in clamp group (p=0.04). No statistically significant difference was found in operative duration, blood loss, positive surgical margins and intra/ peri-operative complications. At three months and one year, renal function was better preserved in non-clamp group compared to clamp group (p=0.001 and 0.007 respectively).

**Conclusion:**

Off clamp open partial nephrectomy is safe and feasible option leading to less decline in renal function.

## INTRODUCTION

Nephron sparing surgery is traditionally used in patients with tumor in anatomically or functionally solitary kidney, bilateral synchronous tumors or in the presence of medical co-morbid condition(s) affecting the renal function.^1^ Open partial nephrectomy (PN) is regarded as ‘standard of care’ procedure for T1 localized renal tumors^2^ & has shown to produce similar oncological but superior functional outcome compared to radical nephrectomy.^3^-^5^ Performing complete tumor excision and achieving adequate hemostasis makes partial nephrectomy a challenging procedure.^6^ Conventional technique for PN involves clamping of renal artery and vein, by providing a clear (blood less) field with reduced renal parenchymal turgor, helping in precise tumor removal, achieving hemostasis and adequate repair of renal parenchyma and collecting system. Studies have shown that each minute of ischemia is crucial in determining renal damage.^7^ Various methods have been described in the literature to introduce techniques to limit or eliminate warm ischemia time (WIT).^7^ These include complete clampless, only renal artery clamping, super selective clamping, use of cold ischemia and induced hypotension.

This study aims to investigate the feasibility and oncological and functional outcome of off-clamp vs. hilar clamping open partial nephrectomy in terms of serum creatinine and e-GFR.

## METHODS

This is a retrospective cohort study of patients who underwent unilateral, open partial nephrectomy for renal tumors between January 2009 to December 2016 at our institution. An Institutional Review Board (IRB) approval was obtained (4034-SUR-ERC-16).

All adult patients (≥18 years) with normal pre-operative renal function (serum Creatinine ≤1.3 mg/dl and estimated glomerular filtration rate (eGFR) of ≥60 ml/min/1.73m^2^ who underwent unilateral, open partial nephrectomy for solitary renal tumor suspicious of malignancy on pre-operative CT scan with at-least one year follow up were included. We excluded patients with tumor in solitary kidney or those who required intra-operative conversion to radical nephrectomy.

The variables studied included patients’ demographics such as age, gender, weight, height, body mass index (BMI), co-morbid medical condition(s) such as diabetes mellitus (DM) and hypertension (HTN), American society of anesthesiologist (ASA) score, tumor characteristics like tumor size and location, laterality, R.E.N.A.L nephrometry score 8 {comprising of **R**adius (tumor size and maximum dimension), **E**xophytic or endophytic nature, **N**earness of tumor to collecting system, **A**nterior or posterior and **L**ocation relative to polar lines}, intra-operative data such as overall operative time, warm ischemia time, estimated blood loss and transfusion rate, post-operative outcomes such as length of hospital stay, tumor histology, surgical margins, Fuhrman’s nuclear grade and 30-days post-operative complications measured by modified Clavian-dindo classification. Patients’ renal function i.e. serum Creatinine and eGFR were assessed pre-operatively, at three and 12 months follow up.

### Surgical technique

The decision to perform renal hilar clamping vs. off clamp surgery was at individual surgeons’ discretion using either retroperitoneal supra 12^th^ flank or trans-peritoneal approach. A cuff of fat overlying the tumor was preserved for correct pathological staging.

**Fig.1:**
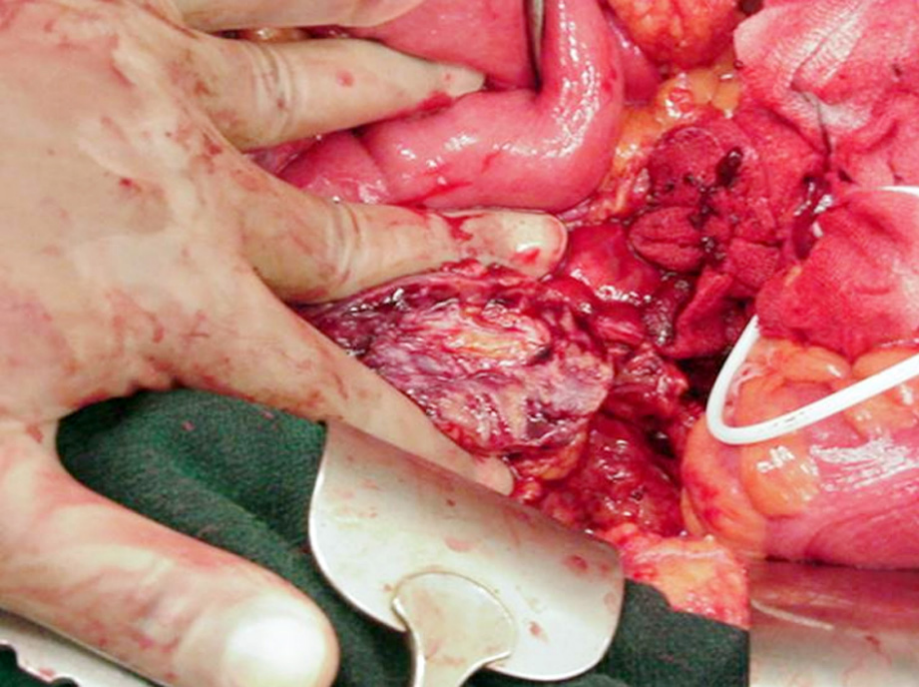
Technique of manual renal parenchymal compression. The cut surface of renal parenchyma can be seen compressed b/w assistant’s fingers (transperitoneal approach).

In case of hilar clamping, vascular bulldog clamps were applied to renal artery and vein after dissection of renal hilum and tumor excision was accomplished. In case of off-clamp procedure, a manual parenchymal compression using fingers to circumscribe the tumor and hence bleeding control was done (Fig.1). The tumor excision was completed using combination of electrocautry, sharp and blunt dissection keeping a few millimeter of renal parenchyma around the tumor. Four quadrant tumor bed biopsy were also sent for frozen section analysis.

Renorrhaphy was performed by suturing the renal bed using 2-0 vicryl suture with intervening oxidized cellulose Surgiceal™ bolsters to approximate the renal parenchyma. We did not use intra-operative ultrasound to determine the margins of tumor, or any sealant device like argon beam etc. nor did we put ice slush on kidney surface for cold ischemia.

### Follow up

All patients were followed up at three, six and 12 months and thereafter every six months. Abdominal ultrasound was done at three months follow up while CT scan at six months and thereafter annually. We analyzed difference in renal function (eGFR) between the two groups at different points i.e. pre-operatively, immediate post-operative, three months and one year.

### Statistical analysis

The data was analyzed on SPSS version 22 and Stata software. The variables were compared between off-clamp and hilar clamp groups. The student t- test was used for continuous variables and chi-square test, Fischer exact test and Mann Whitney U test were applied for categorical variables. A p-value of < 0.05 was considered to be significant.

The primary outcome of our study was to determine the difference in renal function between two groups at short and long term while the secondary outcome was to compare the intra-operative and post-operative variables such as positive surgical margins, blood loss, operative time etc.

## RESULTS

A total of 90 partial nephrectomies were performed out of which 65 cases fulfilling the inclusion criteria were included in final analysis. Off-clamp procedure was done in 43 patients while hilar clamp was applied in 22 patients. Patients’ demographics and pre-operative factors are shown in Table-I.

**Table-I:** Patients demographics and pre-oprative characteristics.

Patient characteristics	Group-1 Hilar clamp (n= 22)	Group-2 Off-clamp (n=43)	p-value
Mean age, years (SD)	56.23 ± 10.23	54.0 ± 10.9	0.44
*Gender*			
Male	13	31	0.29
Female	9	12	
BMI, kg/m^2^, mean (SD)	26.23 ± 3.69	26.1 ± 4.25	0.97
Hypertension, no. (%)	14 (64)	26 (60)	0.8
DM, no. (%)	14 (64)	22 (51)	0.34
HTN + DM, no. (%)	9 (41)	16 (37)	0.77
*ASA, no. (%)*			
I	4 (18)	12 (28)	0.615
II	11 (50)	21 (49)	
III	7 (32)	10 (23)	

Both the groups were comparable with regard to age, gender, BMI, ASA, co-morbid medical conditions and pre-operative renal function. With regard to tumor characteristics, a significantly higher proportion of off-clamp procedures were done on right kidney compared to left kidney (p=0.03). With respect to complexity of tumor measured by R.E.N.A.L nephrometry score, the tumor in hilar clamping group were more complex (mean score 7.05 ± 1.7) compared to off clamp group (mean score 6.1 ± 1.5) (p =0.04). This was mainly due to larger mean size 4.71 ± 1.31 cm in hilar clamp group compared to off-clamp partial nephrectomy with mean size 3.81 ± 1.0 cm (p=0.003) (Table-II). No significant difference was found in other parameters of R.E.N.A.L nephrometry score.

**Table-II:** Tumor characteristics.

Variable	Group-1 Hilar clamp (n= 22)	Group-2 Off-clamp (n=43)	p-value
*Tumor laterality*			
Right, no. (%)	7 (32)	26 (60)	0.03
Left, no. (%)	15 (64)	17 (40)	
Tumor diameter on CT (cm), Mean (SD)	4.71 ± 1.31	3.81 ± 1.0	0.003
R.E.N.A.L nephrometry score, Mean (SD)	7.05 ± 1.76	6.16 ± 1.52	0.04
*Tumor Histopathology, no. (%)*			
Clear cell RCC	17 (77)	31 (71)	0.183
Papillary RCC	3 (14)	8 (19)	
Chromophobe RCC	0	2 (5)	
Oncocytoma	0	2 (5)	
Angiomyolipoma	2 (9)	0	
*Tumor Fuhrman’s grade (from 61 cases), no. (%)*			
1	3 (15)	10 (24)	0.7
2	14 (70)	26 (64)	
3	3 (15)	5 (12)	
Tumor diameter Pathological (cm), Mean (SD)	4.53 ± 2.01	3.48 ± 0.96	0.03

The mean operative time was comparable in both groups and mean warm ischemia time in hilar clamp group was 19.7 ± 6.1 minutes. The mean estimated blood loss was more in off-clamp group 619 ± 382 mls compared to 525 ± 233 mls in hilar clamp group, however, this difference was not statistically significant (p=0.29) (Table-III). No difference was found in blood transfusion rate b/w the two groups.

**Table-III:** Peri-operative characteristics.

Variable	Group-1 Hilar clamp (n= 22)	Group-2 Off-clamp (n=43)	p-value
Operative time (min), Mean (SD)	193.8 ± 51.7	185.2 ± 52.2	0.53
Warm ischemia time (min), Mean (SD)	19.72 ± 6.16	N/A	--
Estimated blood loss(ml), Mean (SD)	525 ± 233.4	619 ± 382.5	0.29
*Transfusion needed, no. (%)*			
Yes	6 (27)	12 (28)	0.96
No	16 (73)	31 (72)	
*Complications, No. (%)*			
Yes	1 (5)	5 (12)	0.35
No	21 (95)	38 (88)	

Pathological data revealed 4 cases to be benign tumors (two each of angiomyolipoma and oncocytoma), while renal cell carcinoma (RCC) was found in 61 cases (pT1a n= 36 and pT1b n=25). Majority of tumors were clear cell RCC (78.7%) followed by papillary RCC and Chromophobe tumors. Intra-operative frozen section margins and final surgical margins were negative in all patients. No significant difference was found in pathological outcome such as tumor histology or nuclear grade between the two groups. The mean pathological size of tumor was smaller (3.48 ± 0.96 cm) in off-clamp group vs. 4.53 ± 2.0 cms in hilar clamp group (p =0.02) however in both the groups the pathological tumor size was smaller compared to pre-operative radiological size.

At a mean follow up of 25.2 months, ipsilateral recurrence was identified in two patients with clear cell pathology (one in each clamp and off-clamp group) who were subsequently managed by radical nephrectomy. Distant metastasis was found in one case after 38 months and the overall survival was 87.7%.

**Fig.2:**
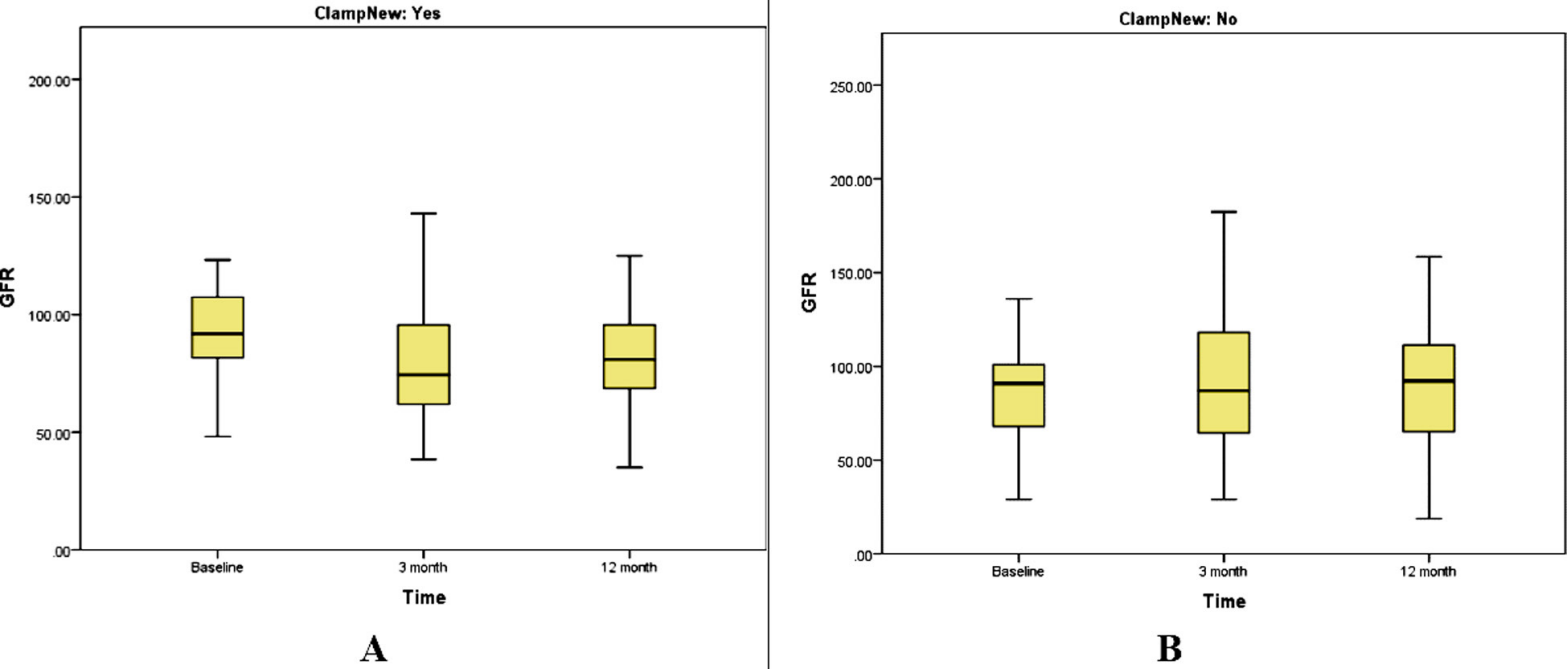
Comparison of eGFR at baseline, 3 months and 12 months. A) Hilar clamp group, B) Off-clamp group.

We found a profound decline in eGFR in hilar clamp group at three months from 96.2 ± 34.2 to 80.7 ± 30.8 which later increased to 84.4 ± 28.6 ml/min/1.73 m^2^ at one-year period, however in off-clamp group, eGFR surprisingly tended to rise from pre-operative 88.0 ± 36.11 to 93.14 ± 40.7 ml/min/1.73 m^2^ at three months that later declined to 91.93 ± 39.6 ml/min/1.73 m^2^ at one year (Table-IV) (Fig.2).We didn’t measure association of age, gender, co-morbid medical conditions and tumor size with change in renal function in two groups due to small sample size not adequate enough for multivariate analysis.Using non-parametric test (Mann Whitney U test), the difference in eGFR (pre-op vs. three months’ post op) and pre-op vs. 12 months’ post op was found to be significantly different (p-values 0.001 and 0.007- respectively) b/w off-clamp and hilar clamping groups.

**Table-IV:** Renal function evaluation.

Variable	Group-1 Hilar clamp (n= 22)	Group-2 Off-clamp (n=43)	p-value
Pre-operative eGFR (ml/min per 1.73 m^2^), Mean (SD)	96.2 ± 34.2	88.00 ± 36.1	0.37
3-month eGFR (ml/min per 1.73 m^2^), Mean (SD)	80.7 ± 30.8	93.1 ± 40.7	0.17
12 months eGFR (ml/min per 1.73 m^2^), Mean (SD)	84.4 ± 28.63	91.9 ± 39.6	0.38

The complications were seen in six patients which were mainly clavian grade two and three including prolonged ileus in two patients, chest infection in two & urine leakage requiring stent placement in two patients. However, these were statistically insignificant between two groups (Table-III).

## DISCUSSION

Despite introduction of minimally invasive surgery over the last two decades, open partial nephrectomy still retains its role for the treatment of renal tumor with the largest worldwide clinical experience and considered as reference gold standard for PN by American urological association guidelines.^8^ The aim of PN is not only cancer control but maximum renal functional preservation as well.

Renal hilum clamping exposes remaining nephrons to ischemia-reperfusion injury.^9^ Conventional NSS comprises of dissection of renal pedicle (artery and vein) with clamping (warm ischemia) and cooling (cold ischemia) to gain better bleeding control.^10^

Multiple factors could affect renal function after partial nephrectomy i.e. baseline kidney quality, associated medical comorbid conditions, preserved renal parenchymal volume and duration of warm ischemia.^4^ Among these factors, the duration of ischemia is the surgically modifiable factor.^9^ Prior reports, favors off clamp technique for better preservation of renal function.^11^-^14^ However, data on long-term functional outcome is sparse. Hung et al.^12^ reported that the “trifecta” of partial nephrectomy (i.e. -ve surgical margin, reno-protection and lower complications) are better preserved in off-clamp technique.

Various methods have been described in literature as an alternate to renal hilar control such as manual compression,^15^ cable-tie devices,^16^,^17^ use of soft bowel clamp or resection using ‘hemostatic’ energy sources.^18^,^19^ These techniques eliminate or minimize warm ischemia thus potentially protecting the function of non-tumor bearing kidney. Manual compression of renal parenchyma can maintain a reno-protective effect and also reduce the risk of renal pedicle vascular injury.^20^ Mcjean et al.^21^ described manual clamping & reported that it reduces the global ischemia to kidney but consequences like greater blood loss and positive surgical margins are main concerns. Use of hilar clamping enables improved visualization kidney repair using relatively bloodless field.^22^

The impact of surgical technique (hilar clamping vs. off clamp) on post-operative renal function preservation is debatable as large multi-institutional studies and randomized controlled trials are lacking.

Studies have shown that every minute of ischemia could have significant impact on post-operative renal function and longer warm ischemia can lead to acute renal failure with an odd ratio of 1.05 for each 1-minute increase.^9^ A warm ischemia time of >20-25 minutes is associated with greater risk of acute and chronic renal injury with need of hemodialysis in future.^7^,^9^,^23^-^25^ In our study, the hilar clamping group had WIT of 19 minutes which is considerably lower compared to other studies. Cheng et al.^23^ noted significant deterioration in eGFR for hilar clamping compared to selective renal parenchymal clamping but this benefit did not translate into long-term (90 days) renal function improvement.

To our knowledge, this is the first report from Pakistan showing better long term functional outcome of off-clamp partial nephrectomy. Our mean tumor size and nephrometry scores reflect moderately complex cases. We did not perform simple enucleation of tumors rather few mm margin was left all around the pseudo capsule of tumor, leading no positive surgical margins but the EBL was slightly higher (though insignificant) in off-clamp group. We found significantly better long-term functional outcome in off-clamp group.There are few shortcomings in our paper. It was a single center, retrospective analysis of small cohort of patients. We did not have a strictly defined indication to perform off-clamp partial nephrectomy and it was dependent on size and location of tumor, patients’ characteristics and surgeons’ preference.

Although both cohort of patients was relatively similar (matched) with respect to comorbid medical conditions, age etc., we could not determine the other confounding factors individually on multivariate analysis due to small sample size.

We measured and compared radiological and pathological size of tumor between two groups but did not measure volume of the remaining kidney or volume loss as a result of partial nephrectomy and hence could not correlate the functional outcome with the renal volume loss.

### Limitations of the study

Renal functional outcome was evaluated solely on the basis of changes in post-operative eGFR. The impact of off –clamp partial nephrectomy on the kidney can also be determined by other methods which evaluate split renal function such as nuclear scans.

## CONCLUSION

Our results support that off-clamp partial nephrectomy is a safe procedure which can be performed in select group of patients with comparable oncological outcome and better long term functional outcome compared to hilar clamping.

### Authors’ Contribution:

**FA and SMN:** Conceived, designed and did statistical analysis & editing of manuscript.

**IK, SMN and MA:** Did data collection and manuscript writing.

**IK:** Takes the responsibility and is accountable for all aspects of the work in ensuring that questions related to the accuracy or integrity of any part of the work are appropriately investigated and resolved.
